# Bis(2-methyl­piperidinium) penta­chlorido­anti­monate(III)

**DOI:** 10.1107/S1600536812017163

**Published:** 2012-04-25

**Authors:** Qian Xu

**Affiliations:** a Ordered Matter Science Research Center, College of Chemistry and Chemical Engineering, Southeast University, Nanjing 211189, People’s Republic of China

## Abstract

The asymmetric unit of the title compound, (C_6_H_14_N)_2_[SbCl_5_], contains one cation and half of the anion on a special position (specifically, the Sb^III^ ion and three chloride anions are situated on a mirror plane). In the [SbCl_5_]^2−^ unit, the Sb^III^ ion is coordinated by five chloride anions [Sb—Cl = 2.3721 (11)–2.6656 (12) Å] in a distorted square-pyramidal geometry. However, one chloride anion from a neighbouring [SbCl_5_]^2−^ unit provides a short Sb⋯Cl contact of 3.3600 (12) Å and completes the Sb coordination environment up to an elongated octa­hedron. In the crystal, N—H⋯Cl hydrogen bonds link cations and anions into columns propagating along [100].

## Related literature

For the crystal structure of bis­(4-benzyl­piperidinium) penta­chloridoanti­monate(III), see: Marsh (1995 ▶). For background to ferroelectric metal-organic frameworks, see: Fu *et al.* (2009 ▶); Ye *et al.* (2006 ▶); Zhang *et al.* (2008 ▶, 2010 ▶).
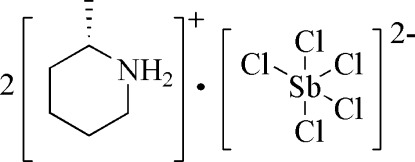


## Experimental

### 

#### Crystal data

(C_6_H_14_N)_2_[SbCl_5_]
*M* *_r_* = 499.36
Orthorhombic, 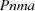

*a* = 7.5995 (15) Å
*b* = 23.165 (5) Å
*c* = 11.453 (2) Å
*V* = 2016.2 (7) Å^3^
*Z* = 4
Mo *K*α radiation
μ = 2.03 mm^−1^
*T* = 293 K
0.28 × 0.25 × 0.21 mm

#### Data collection

Rigaku Mercury70 CCD diffractometer
Absorption correction: multi-scan (*CrystalClear*; Rigaku, 2005 ▶) *T*_min_ = 0.421, *T*_max_ = 0.558
19754 measured reflections
2361 independent reflections
1938 reflections with *I* > 2σ(*I*)
*R*_int_ = 0.061

#### Refinement

*R*[*F* ^2^ > 2σ(*F* ^2^)] = 0.033
*wR*(*F* ^2^) = 0.065
*S* = 1.08
2361 reflections
98 parameters
H-atom parameters constrained
Δρ_max_ = 0.58 e Å^−3^
Δρ_min_ = −0.52 e Å^−3^

### 

Data collection: *SCXmini Benchtop Crystallography System Software* (Rigaku, 2006 ▶); cell refinement: *SCXmini Benchtop Crystallography System Software*; data reduction: *SCXmini Benchtop Crystallography System Software*; program(s) used to solve structure: *SHELXS97* (Sheldrick, 2008 ▶); program(s) used to refine structure: *SHELXL97* (Sheldrick, 2008 ▶); molecular graphics: *DIAMOND* (Brandenburg & Putz, 2005 ▶); software used to prepare material for publication: *SHELXL97*.

## Supplementary Material

Crystal structure: contains datablock(s) I, global. DOI: 10.1107/S1600536812017163/cv5279sup1.cif

Structure factors: contains datablock(s) I. DOI: 10.1107/S1600536812017163/cv5279Isup2.hkl

Additional supplementary materials:  crystallographic information; 3D view; checkCIF report

## Figures and Tables

**Table 1:** Hydrogen-bond geometry (Å, °)

*D*—H⋯*A*	*D*—H	H⋯*A*	*D*⋯*A*	*D*—H⋯*A*
N1—H1*D*⋯Cl3^i^	0.90	2.40	3.226 (3)	153
N1—H1*E*⋯Cl1^ii^	0.90	2.48	3.373 (3)	173

Symmetry codes: (i) 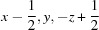; (ii) 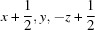.

**Fig. 1.**
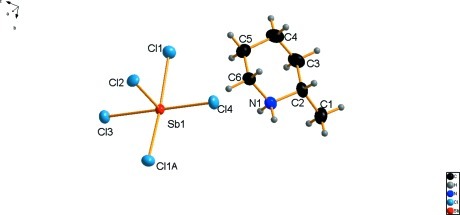
The molecular structure of the (I), with the displacement ellipsoids drawn at the 30% probability level [symmetry code: (A) x, 0.5 - y, z].

**Fig. 2.**
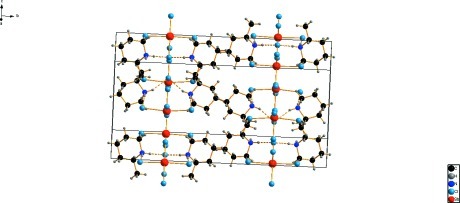
A portion of the packing diagram with hydrogen bonds shown as dashed lines.

## supplementary crystallographic information

### Comment

As a contribution to a search for new ferroelectric materials (Fu *et al.*, 2009; Ye *et al.*, 2006; Zhang *et al.*, 2008; Zhang *et al.*, 2010), we have synthesized the title compound, (I). Herewith we present its crystal structure.

The asymmetric unit of (I), 2(C_6_H_14_N)^+^[SbCl_5_]^2-^, contains one cation and one-half of the anion in a special position (Fig. 1). The Sb1 atoms coordinated in a slightly distorted square-pyramidal geometry by five Cl atoms and distance of the top Cl2 and Sb1 is 2.3721 (11) Å much shorter than the mean values of other Sb—Cl[2.636 (11) Å]. The bond angles around the Sb1 are in the range 84.93 (2)–91.454 (19)° and correspond to those observed in the related compound 2(C_12_H_18_N)^+^[SbCl_5_]^2-^ (Marsh, 1995).

In the crystal structure, intermolecular N—H···Cl hydrogen bonds (Table 1) link cations and anions into columns propagated in [100] (Fig. 2). In the title compound, no dielectric anomalies were observed in the range from 190 K to its melting point, which is more than 357 K.

### Experimental

The mixture of SbCl_3_(1.1 g, 5 mmol) and 2-methypiperidine (1.05 g, 10 mmol) in hydrochloric acid solution was stirred for several minutes at room temperature. Colourless crystals suitable for X-ray diffraction analysis were obtained by slow evaporation of the solution at room temperature over 2 weeks.

### Refinement

All H atoms were placed in geometrically idealized positions and constrained to ride on their parent atoms with C—H = 0.93–0.98 Å and N—H = 0.90 Å, and with *U*_iso_(H) = 1.2–1.5 *U*_iso_(C, N).

### Crystal data

**Table:**

(C_6_H_14_N)_2_[SbCl_5_]	*F*(000) = 1000
*M**_r_* = 499.36	*D*_x_ = 1.645 Mg m^−^^3^
Orthorhombic, *P**n**m**a*	Mo *K*α radiation, λ = 0.71073 Å
Hall symbol: -P 2ac 2n	Cell parameters from 2361 reflections
*a* = 7.5995 (15) Å	θ = 2.2–27.5°
*b* = 23.165 (5) Å	µ = 2.03 mm^−^^1^
*c* = 11.453 (2) Å	*T* = 293 K
*V* = 2016.2 (7) Å^3^	Block, colourless
*Z* = 4	0.28 × 0.25 × 0.21 mm

### Data collection

**Table:**

Rigaku Mercury70 CCD diffractometer	2361 independent reflections
Radiation source: fine-focus sealed tube	1938 reflections with *I* > 2σ(*I*)
Graphite monochromator	*R*_int_ = 0.061
ω scans	θ_max_ = 27.5°, θ_min_ = 3.2°
Absorption correction: multi-scan (*CrystalClear*; Rigaku, 2005)	*h* = −9→9
*T*_min_ = 0.421, *T*_max_ = 0.558	*k* = −30→30
19754 measured reflections	*l* = −14→14

### Refinement

**Table:**

Refinement on *F*^2^	Primary atom site location: structure-invariant direct methods
Least-squares matrix: full	Secondary atom site location: difference Fourier map
*R*[*F*^2^ > 2σ(*F*^2^)] = 0.033	Hydrogen site location: inferred from neighbouring sites
*wR*(*F*^2^) = 0.065	H-atom parameters constrained
*S* = 1.08	*w* = 1/[σ^2^(*F*_o_^2^) + (0.0181*P*)^2^ + 1.8699*P*] where *P* = (*F*_o_^2^ + 2*F*_c_^2^)/3
2361 reflections	(Δ/σ)_max_ = 0.001
98 parameters	Δρ_max_ = 0.58 e Å^−^^3^
0 restraints	Δρ_min_ = −0.52 e Å^−^^3^

### Special details

**Table:**

Geometry. All e.s.d.'s (except the e.s.d. in the dihedral angle between two l.s. planes) are estimated using the full covariance matrix. The cell e.s.d.'s are taken into account individually in the estimation of e.s.d.'s in distances, angles and torsion angles; correlations between e.s.d.'s in cell parameters are only used when they are defined by crystal symmetry. An approximate (isotropic) treatment of cell e.s.d.'s is used for estimating e.s.d.'s involving l.s. planes.
Refinement. Refinement of *F*^2^ against ALL reflections. The weighted *R*-factor *wR* and goodness of fit *S* are based on *F*^2^, conventional *R*-factors *R* are based on *F*, with *F* set to zero for negative *F*^2^. The threshold expression of *F*^2^ > σ(*F*^2^) is used only for calculating *R*-factors(gt) *etc*. and is not relevant to the choice of reflections for refinement. *R*-factors based on *F*^2^ are statistically about twice as large as those based on *F*, and *R*- factors based on ALL data will be even larger.

### Fractional atomic coordinates and isotropic or equivalent isotropic displacement parameters (Å^2^)

**Table:**

	*x*	*y*	*z*	*U*_iso_*/*U*_eq_	
C1	0.8199 (5)	0.1245 (2)	−0.1957 (3)	0.0703 (12)	
H1A	0.7817	0.1631	−0.2129	0.105*	
H1B	0.7841	0.0991	−0.2575	0.105*	
H1C	0.9457	0.1238	−0.1887	0.105*	
C2	0.7387 (5)	0.10494 (17)	−0.0830 (3)	0.0554 (10)	
H2	0.6106	0.1081	−0.0900	0.066*	
C3	0.7834 (6)	0.04338 (18)	−0.0511 (4)	0.0750 (13)	
H3A	0.7347	0.0177	−0.1098	0.090*	
H3B	0.9103	0.0388	−0.0519	0.090*	
C4	0.7143 (7)	0.0260 (2)	0.0672 (4)	0.0866 (15)	
H4A	0.7536	−0.0128	0.0855	0.104*	
H4B	0.5866	0.0259	0.0659	0.104*	
C5	0.7785 (6)	0.06724 (18)	0.1599 (4)	0.0707 (12)	
H5A	0.7284	0.0566	0.2348	0.085*	
H5B	0.9055	0.0647	0.1661	0.085*	
C6	0.7275 (5)	0.12723 (16)	0.1310 (3)	0.0552 (10)	
H6A	0.7741	0.1534	0.1895	0.066*	
H6B	0.6002	0.1306	0.1314	0.066*	
N1	0.7977 (4)	0.14369 (12)	0.0122 (3)	0.0499 (7)	
H1D	0.7632	0.1799	−0.0044	0.060*	
H1E	0.9160	0.1435	0.0147	0.060*	
Cl1	0.73805 (11)	0.13613 (4)	0.45405 (8)	0.0508 (2)	
Cl2	0.58634 (15)	0.2500	0.61136 (10)	0.0463 (3)	
Cl3	1.05345 (15)	0.2500	0.57047 (11)	0.0489 (3)	
Cl4	0.48085 (16)	0.2500	0.31467 (11)	0.0529 (3)	
Sb1	0.76256 (3)	0.2500	0.44041 (2)	0.03097 (9)	

### Atomic displacement parameters (Å^2^)

**Table:**

	*U*^11^	*U*^22^	*U*^33^	*U*^12^	*U*^13^	*U*^23^
C1	0.070 (3)	0.095 (3)	0.046 (2)	−0.005 (2)	−0.001 (2)	−0.007 (2)
C2	0.047 (2)	0.065 (2)	0.054 (2)	0.0012 (18)	−0.0107 (18)	−0.0133 (18)
C3	0.090 (3)	0.048 (2)	0.088 (3)	−0.004 (2)	0.006 (3)	−0.017 (2)
C4	0.111 (4)	0.051 (3)	0.098 (4)	−0.014 (3)	0.008 (3)	−0.005 (3)
C5	0.077 (3)	0.071 (3)	0.064 (3)	−0.007 (2)	−0.003 (2)	0.012 (2)
C6	0.065 (2)	0.051 (2)	0.050 (2)	−0.0070 (19)	0.0054 (19)	−0.0070 (17)
N1	0.0522 (17)	0.0429 (17)	0.0547 (18)	0.0026 (14)	−0.0054 (15)	−0.0050 (14)
Cl1	0.0496 (5)	0.0453 (5)	0.0576 (5)	0.0051 (4)	0.0006 (4)	−0.0039 (4)
Cl2	0.0521 (7)	0.0532 (7)	0.0336 (6)	0.000	0.0144 (5)	0.000
Cl3	0.0438 (6)	0.0535 (7)	0.0493 (7)	0.000	−0.0117 (6)	0.000
Cl4	0.0481 (7)	0.0584 (8)	0.0523 (7)	0.000	−0.0189 (6)	0.000
Sb1	0.02942 (15)	0.03721 (16)	0.02627 (14)	0.000	0.00072 (12)	0.000

### Geometric parameters (Å, º)

**Table:**

C1—C2	1.500 (5)	C5—C6	1.480 (5)
C1—H1A	0.9600	C5—H5A	0.9700
C1—H1B	0.9600	C5—H5B	0.9700
C1—H1C	0.9600	C6—N1	1.511 (5)
C2—N1	1.481 (4)	C6—H6A	0.9700
C2—C3	1.511 (6)	C6—H6B	0.9700
C2—H2	0.9800	N1—H1D	0.9000
C3—C4	1.509 (6)	N1—H1E	0.9000
C3—H3A	0.9700	Cl1—Sb1	2.6491 (10)
C3—H3B	0.9700	Cl2—Sb1	2.3721 (11)
C4—C5	1.510 (6)	Cl3—Sb1	2.6656 (12)
C4—H4A	0.9700	Cl4—Sb1	2.5802 (12)
C4—H4B	0.9700	Sb1—Cl1^i^	2.6491 (10)
			
C2—C1—H1A	109.5	C4—C5—H5A	109.5
C2—C1—H1B	109.5	C6—C5—H5B	109.5
H1A—C1—H1B	109.5	C4—C5—H5B	109.5
C2—C1—H1C	109.5	H5A—C5—H5B	108.1
H1A—C1—H1C	109.5	C5—C6—N1	110.3 (3)
H1B—C1—H1C	109.5	C5—C6—H6A	109.6
N1—C2—C1	109.0 (3)	N1—C6—H6A	109.6
N1—C2—C3	109.0 (3)	C5—C6—H6B	109.6
C1—C2—C3	113.6 (3)	N1—C6—H6B	109.6
N1—C2—H2	108.4	H6A—C6—H6B	108.1
C1—C2—H2	108.4	C2—N1—C6	113.8 (3)
C3—C2—H2	108.4	C2—N1—H1D	108.8
C4—C3—C2	113.0 (4)	C6—N1—H1D	108.8
C4—C3—H3A	109.0	C2—N1—H1E	108.8
C2—C3—H3A	109.0	C6—N1—H1E	108.8
C4—C3—H3B	109.0	H1D—N1—H1E	107.7
C2—C3—H3B	109.0	Cl2—Sb1—Cl4	89.56 (5)
H3A—C3—H3B	107.8	Cl2—Sb1—Cl1	84.93 (2)
C3—C4—C5	110.5 (4)	Cl4—Sb1—Cl1	88.54 (2)
C3—C4—H4A	109.6	Cl2—Sb1—Cl1^i^	84.93 (2)
C5—C4—H4A	109.6	Cl4—Sb1—Cl1^i^	88.542 (19)
C3—C4—H4B	109.6	Cl1—Sb1—Cl1^i^	169.47 (4)
C5—C4—H4B	109.6	Cl2—Sb1—Cl3	90.40 (4)
H4A—C4—H4B	108.1	Cl4—Sb1—Cl3	179.96 (4)
C6—C5—C4	110.6 (4)	Cl1—Sb1—Cl3	91.454 (19)
C6—C5—H5A	109.5	Cl1^i^—Sb1—Cl3	91.454 (19)
			
N1—C2—C3—C4	53.2 (5)	C4—C5—C6—N1	−56.7 (5)
C1—C2—C3—C4	175.0 (4)	C1—C2—N1—C6	−178.6 (3)
C2—C3—C4—C5	−55.2 (6)	C3—C2—N1—C6	−54.0 (4)
C3—C4—C5—C6	56.5 (5)	C5—C6—N1—C2	57.2 (4)

Symmetry code: (i) *x*, −*y*+1/2, *z*.

### Hydrogen-bond geometry (Å, º)

**Table:**

*D*—H···*A*	*D*—H	H···*A*	*D*···*A*	*D*—H···*A*
N1—H1*D*···Cl3^ii^	0.90	2.40	3.226 (3)	153
N1—H1*E*···Cl1^iii^	0.90	2.48	3.373 (3)	173

Symmetry codes: (ii) *x*−1/2, *y*, −*z*+1/2; (iii) *x*+1/2, *y*, −*z*+1/2.
